# TRS-PCR profiles correlate with polymorphisms of the genomic *o454-nlpD* region, virulence factors repertoire, and phylogenetic groups among uropathogenic *Escherichia coli* strains isolated from patients from Lodz region, Poland

**DOI:** 10.1186/s13099-024-00603-1

**Published:** 2024-02-23

**Authors:** Anna B. Kubiak-Szeligowska, Marta Majchrzak, Pawel Parniewski

**Affiliations:** Institute of Medical Biology, Polish Academy of Sciences, 106 Lodowa Str., 93-232 Lodz, Poland

**Keywords:** Uropathogenic *Escherichia coli*, TRS-PCR profiling, *o454-nlpD* genomic polymorphism, Virulence factors

## Abstract

Extraintestinal urinary tract infections are mainly caused by uropathogenic strains of *E. coli*. UPECs are a heterogeneous group of strains possessing various genes associated with virulence traits. It was demonstrated that changes in the composition of the *o454-nlpD* region and genetic variation in the *mutS-rpoS* chromosomal region in ExPEC strains are correlated with their virulence, particularly in those with the pattern III *o454-nlpD* region and belonging to phylogenetic group B2. In this study, we investigated the presence and distribution of the *o454-nlpD* genomic polymorphism in our collection of 124 uropathogenic *E. coli* strains, examining the correlation of *o454-nlpD* region types with the virulence factors studied. Our findings revealed a positive association between certain virulence factors in UPEC strains and the presence of pattern III in the *o454-nlpD* region. Additionally, all these strains were classified under phylogenetic group B2. We also showed that the highly pathogenic group of *E. coli* identified by examining the polymorphism of the *o454-nlpD* region coincides with the highly pathogenic group of uropathogens we identified in the averaged TRS-PCR analysis.

**Supplementary Information:**

The online version contains supplementary material available at 10.1186/s13099-024-00603-1.

## Introduction

Extraintestinal infections due to *Escherichia coli* strains (ExPEC) pose a threat to public health worldwide. The broad spectrum of diseases they cause and the increasing costs associated with morbidity and mortality constitute a growing significant epidemic problem [1].

ExPEC is a highly heterogeneous group of strains with a wide spectrum of virulence factors and high flexibility in adapting to various environments, which enables and leads to the initiation of infection when an immunocompetent host is encountered [2–6].

Uropathogenic *E. coli* (UPEC) strains cause the vast majority of urinary tract infections (UTIs), classified as lower or upper and uncomplicated or complicated [7]. Moreover, UPEC are specialized bacteria owing to virulence and physiological factors that enable them to adapt to diverse environments and nutrient availability, which plays a significant role in their pathogenesis [8, 9]. Therefore, Köhler and Dobrindt [6], Foxman [7], and Mann et al. [8] proposed a classification of UPEC based on the site of isolation (urine) and the detection of two virulence-related genes typical of this pathotype. Many virulence factors may occur in different *E. coli* pathotypes of that species, which affects not only the virulence potential of one strain but also the mechanisms of pathogenesis [3, 10]. UPEC strains are very rich in virulence-associated factors. With their vast range of lipopolysaccharides, polysaccharide capsules, toxins, invasins, proteases, and adhesiveness features, they can enter and colonize the urinary tract and further disseminate in the human body [3, 11]. A multiplex-PCR test was created in our laboratory to identify some UPEC virulence genes [12]. Detection of six selected genes was proper to determine the distribution of UPEC-specific genes encoding S fimbriae (*sfaD/sfaE*), P fimbriae (*papC*), α-hemolysin (*hlyA*), cytotoxic necrotizing factor 1 (*cnf1*), uropathogenic-specific protein (*usp*), and the *fimG/fimH* region encoding subunits of type 1 fimbriae frequently present in many *E. coli* pathotypes [12]. Notably, this was a test that detected six factors among many others, such as siderophore systems—yersiniabactin (*fyuA*), salmochelin (*iroN*), aerobactin (*iutA*) or serine-protease autotransporter toxins—Sat (secreted autotransporter toxin), Tsh (temperature-sensitive hemagglutinin), and Pic (protease involved in colonization) [5, 8, 11, 13–20].

There is much evidence that some regions in the *E. coli* genome, such as the *mutS-rpoS* chromosomal region, may constitute a valuable marker for testing virulence potential due to its genetic variability [2, 21–23]. An enormous variability in the *mutS-rpoS* intergenic region and its flanking region revealed that it might change during evolution and HGT (horizontal gene transfer). This variability may contribute to the pathotype-specific polymorphism in this region [2, 23]. The characteristics of the *mutS* gene and the *o454-nlpD* genomic region enabled the association of its variability with some vital *E. coli* features, such as pathotype, virulence factors, and phylogeny [2]. The polymorphism of the *o454-nlpD* region and its correlation with virulence-associated genes may help analyze ExPEC strains [2, 22].

Moreover, this region can be distinguished into a few patterns. One of them—pattern III—was proposed as a marker for identifying highly virulent extraintestinal strains of *E. coli* [2, 24]. This region was also present in the strain designated FHI_NMBU_03, described as a hybrid of ExPEC with pathovars such as UPEC, APEC, NMEC, and IPEC—aEPEC, with virulence factors linked to ETEC [24].

Our study examined the presence and distribution of *o454-nlpD* region genomic polymorphisms in our collection of one hundred and twenty-four uropathogenic *E. coli* strains and the correlation of types of *o454-nlpD* regions with the studied virulence factors. Our aim was also to check whether the highly pathogenic group of *E. coli* identified by examining the polymorphism of the *o454-nlpD* region coincides with the highly pathogenic group of uropathogens identified by us in the averaged TRS-PCR analysis developed in our laboratory [12, 25, 26].

## Materials and methods

### Bacterial strains

The collection of 124 *E. coli* strains isolated from urine was used in this study. All strains were isolated from patients with urinary tract infections between June 2005 and September 2006 (various wards of Military Teaching Hospital No. 2, Medical University of Lodz, Poland). As published elsewhere, this collection of UPEC had virulence factors tested and phylogenetic affiliation specified [25–27]. The collection was serotyped according to the manufacturer’s protocols for *E. coli* (O pool and O single antisera, Statens Serum Institut SSI Diagnostica, Denmark) [26].

### Bacterial growth and genomic DNA isolation

Bacterial growth and genomic DNA isolation for all *E. coli* strains were performed as previously published [26]. Strains from this collection were grown with agitation at 120 RPM overnight at 37 °C in LB liquid broth. A GenElute Bacterial Genomic DNA Kit (Sigma‒Aldrich, St. Louis, MO) was used according to the manufacturer’s protocol to isolate and purify DNA. The concentration and purity ratio in all the samples of the extracted DNA were measured spectrophotometrically (BioPhotometer, Eppendorf, Germany).

### Amplification of the *o454-nlpD* genomic region of *E. coli*

PCR amplification was performed with primers F5 and R2 from Ewers et al. [8] to estimate the size of the *o454-nlpD* region within the tested UPEC collection. The PCR reactions followed the manufacturers’ guidelines for Taq polymerase (Invitrogen™, Life Technologies). Each reaction had a volume of 50 µl, containing 5 µl of 10× PCR buffer, 20 pmol of each primer (F5 and R2), 1.5 mM MgCl_2_, 0.2 mM of each deoxynucleoside triphosphate (dATP, dGTP, dCTP, and dTTP), 1 µl of chromosomal DNA solution (20 ng/μl), and 1 unit of Taq polymerase. The amplification was performed in a T-3000 thermocycler (Biometra, Goettingen, Germany) under customized conditions: initial denaturation for 3 min at 95 °C, 35 cycles: 1 min of denaturation at 95 °C, 1 min of annealing of the primers at 61 °C, 3 min of elongation at 72 °C, and final elongation for 8 min at 72 °C. The amplification products were separated on 1.6% agarose gels in 1× TAE buffer at room temperature until the dye (bromophenol blue) migrated 6 cm from the beginning of the gel (2.4 V/cm). Subsequently, gels were stained with ethidium bromide, photographed, normalized with 100 bp Plus DNA ladder (Fermentas, Thermo Scientific Waltham, MA, USA), and analyzed.

### RFLP analysis of a pattern different A of the *o454-nlpD* region

Restriction digestion of the PCR amplification product containing the unusual *o454-nlpD* pattern (different A), which was approximately 1660 bp in length, was performed with Hae III (Thermo Scientific FastDigest Restriction Enzymes, MA, USA). The whole procedure was performed strictly according to the manufacturer’s protocol. Next, the digestion products were separated on a 1.8% agarose gel on 1× TAE buffer, stained with ethidium bromide, and photographed. The gel was normalized with regard to 1 kb and 50 bp DNA size markers (Fermentas, Thermo Scientific Waltham, Ma, USA).

### TRS-PCR profiling

TRS-PCR profiling was previously developed and performed for the collected *E. coli* strains [12, 25, 26]. For each UPEC strain, similarity dendrograms of CAC-, GTG- and CGG-PCR profiles were generated separately using Pearson correlation (1% optimization, 1% position tolerance), and clustering was performed according to the UPGMA algorithm (BioNumerics software, Applied Maths, Belgium). In this work, we generated an average dendrogram of band profile similarity, showing the grouping of the studied *E. coli* strains. Information regarding the *o454-nlpD* profile, phylotype, and virulence profile was added to the analysis.

## Results

In the initial research stage, we conducted amplification (using F5 and R2 primers) of the *mutS-rpoS* chromosomal region to examine the presence and type of the *o454-nlpD* region in our *E. coli* collection. While performing polymorphism analyses on the *o454-nlpD* region, we discovered an additional pattern with a different length of 1660 bp, differing from the previously described pattern [2]. To identify complementary regions in the tested genomes, we utilized the F5 and R2 primer sequences and the online software available at http://insilico.ehu.es/PCR/. Subsequently, sequences with a length of 1660 bp (representing the atypical *o454-nlpD* pattern) were explicitly chosen to confirm the amplified region’s accuracy.

HaeIII restriction analysis of the 1660 bp PCR products yielded restriction fragments consistent with the predictions from in silico analysis (498, 406, 306, 172, 96, 87, 39, 36, 20 bp, Additional file 1: Fig. S1). Consequently, we verified that the newly identified mutS-rpoS chromosomal region—referred to as “Different A”—corresponds to one of the analyzed *o454-nlpD* patterns.

The pattern distribution of the *o454-nlpD* region for the 124 tested UPEC strains was determined and compared with the phylogroups, virulence factors, and TRS-PCR fingerprints.

**Fig. 1:**
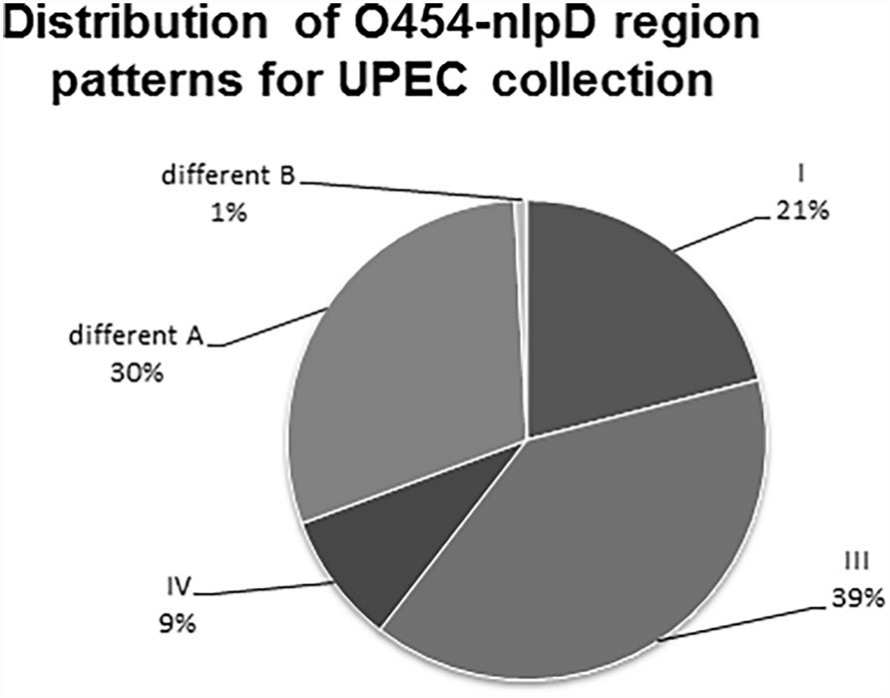
Distribution of *o454-nlpD* region patterns among UPEC strain collections

Pattern III was overrepresented, constituting 39% (49 strains) of the tested isolates. Patterns named different A and pattern I constituted 30% (37 strains) and 21% (26 strains), respectively. Only 9% (11 strains) had pattern IV, and one strain had a different B pattern. Our collection had no strains with pattern II (Fig. 1).

**Fig. 2:**
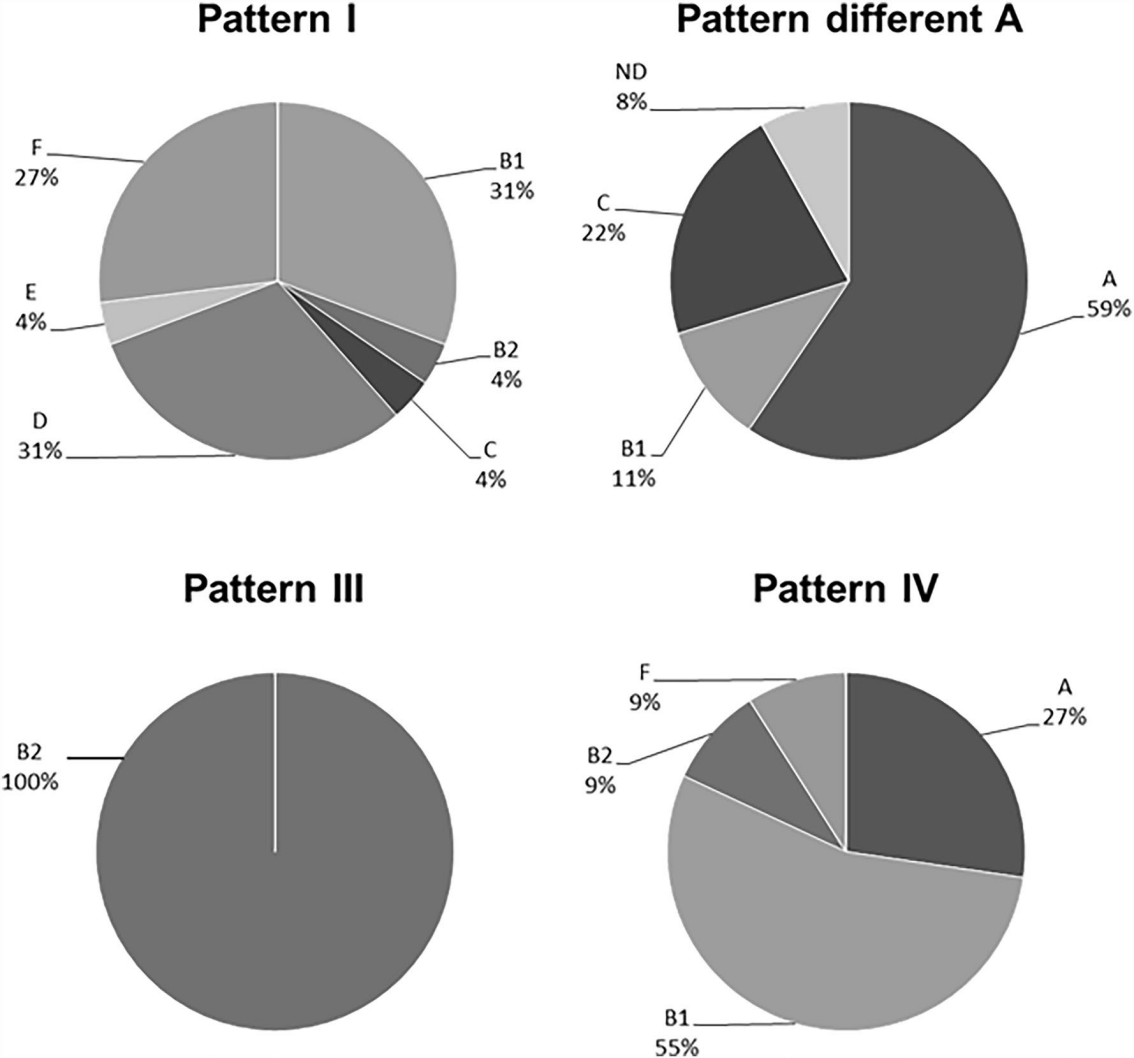
Distribution of phylogroups of UPEC strains with different *o454-nlpD* region patterns

The phylogenetic structure of UPEC strains for obtained *o454-nlpD* region patterns was tested. The results are presented in Fig. 2. The UPEC strains with pattern III all belonged to phylogenetic group B2. The phylogenetic groups D, B1, and F were represented at similar levels among strains with pattern I (31%, 31%, and 27%, respectively). Strains representing phylogenetic group A predominated among strains with a pattern different A (59%). In the case of strains with pattern IV, more than half of this group belonged to phylogenetic group B1 (55%).

Our laboratory UPEC collection of strains had the virulence factor repertoire tested as published elsewhere [12, 26]. The distribution of virulence factors in strains with particular patterns of the *o454-nlpD* region was determined (Table 1). Genes related to siderophore systems, such as *fyuA*, *iutA*, and *iroN*, were detected among all strains with the pattern I. Twenty-five of 26 strains also encoded *fimG/fimH*, one strain had Sat, and two had Tsh—serine-protease autotransporter toxins of Enterobacteriaceae (SPATE). Six strains from this group encoded *papC*, two encoded *sfa*, two encoded *cnf1*, one encoded *usp*, and three encoded *hlyA*.

**Table 1:** Distribution of virulence factors among UPEC strains with particular patterns of the *o454-nlpD* region

Virulence factor	Distribution of *o454-nlpD* patterns			
	Strains with pattern I	Strains with pattern III	Strains with pattern different A	Strains with pattern IV
	[%] (n)	[%] (n)	[%] (n)	[%] (n)
*papC*	23.08 (6)	69.39 (34)	5.41 (2)	9.09 (1)
*sfaD/sfaE*	7.69 (2)	79.59 (39)	2.7 (1)	9.09 (1)
*cnf1*	7.69 (2)	69.39 (34)	2.7 (1)	9.09 (1)
*usp*	3.85 (1)	75.51 (37)	2.7 (1)	9.09 (1)
*hly1*	11.54 (3)	71.43 (35)	2.7 (1)	9.09 (1)
*fimG/fimH*	96.15 (25)	100 (49)	75.68 (28)	72.73 (8)
*fyuA*	38.46 (10)	100 (49)	32.43 (12)	45.45 (5)
*iutA*	53.85 (14)	59.18 (29)	24.33 (9)	72.73 (8)
*iroN*	50 (13)	91.84 (45)	35.14 (13)	81.82 (9)
*sat*	3.85 (1)	28.57 (14)	0 (0)	0 (0)
*tsh*	7.69 (2)	2.04 (1)	2.7 (1)	18.18 (2)
*astA*	19.23 (5)	6.12 (3)	24.33 (9)	27.27 (3)
*aggR*	3.85 (1)	0 (0)	0 (0)	0 (0)
*pic*	3.85 (1)	4.08 (2)	0 (0)	0 (0)
None of tested	3.85 (1)	0 (0)	13.51 (5)	0 (0)
Total number of strains	26	49	11	37

The pattern III strains had at least two virulence profiles associated with uropathogenicity. Moreover, the pattern III strains had the most variable profiles of virulence genes. The gene *fyuA*, which is related to the siderophore system, was detected in all isolates from this group. The genes *iutA*, *iroN*, and sat were present in approximately 59%, 92%, and 29% of isolates, respectively.

Only 2% of strains with the pattern different A possessed the *papC* gene. Each gene, such as *sfaD/sfaE*, *cnf1*, *usp*, and *hlyA*, was present in 1% of these strains. However, genes associated with siderophore systems, such as *fyuA*, *iutA*, *iroN*, as well *astA,* were identified in approximately 32%, 24%, 35%, and 24% of isolates, respectively.

Only three strains with pattern IV encoded the *astA* gene, and one strain encoded the *tsh* gene. Most of them had *iutA* (approx. 73%), *iroN* (approx. 82%), and *fyuA* (approx. 45.5%).

None of the tested strains had virulence genes such as *bfpB*, *invE*, *elt*, *escV*, *stx1*, *stx2*, *estIa*, or *estIb*, and they were not included in statistical analyses associated with the *o454-nlpD* region.

Next, Fisher’s exact test was used to estimate the correlation between the presence of *o454-nlpD* patterns and the identified virulence factors. The relationship was statistically significant when P values were smaller than 0.05. Pattern I: no positive associations were detected; negative association with *cnf1*, *hly1*, *sfaD/sfaE*, and *usp*; no association with *papC*, *fimG/fimH*, *fyuA*, *iutA*, *iroN*, *sat*. Pattern III: positive association with *papC*, *sfaD/sfaE*, *cnf1*, *usp*, *hly1*, *fyuA*, and *iroN*; negative association with *fimG/fimH* and *sat*; no association was observed with *iutA*. Pattern IV: no positive and no negative associations were detected; no association with *papC*, *sfaD/sfaE*, *cnf1*, *usp*, *hly1*, *fimG/fimH*, *iutA*, *fyuA*, *iroN*, and sat. Pattern Different A: no positive associations were detected; negative associations with *papC*, *sfaD/sfaE*, *cnf1*, *usp*, *hly1*, *fimG/fimH*, *fyuA*, *iutA*, *iroN*, and *sat*.

For virulence factors such as *tsh*, *astA*, *aggR*, and *pic*, no association was observed with any tested pattern of the *o454-nlpD* region. Detailed information is gathered in Table 2.

**Table 2:**
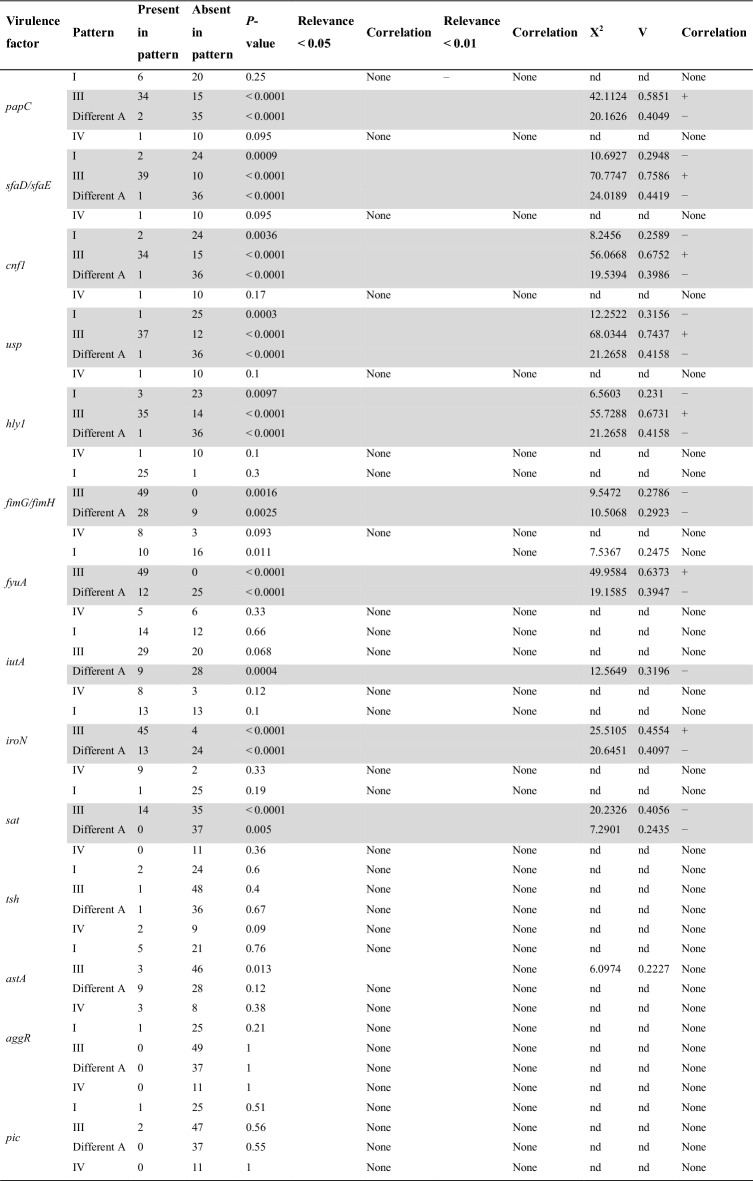
Correlation between particular virulence factors and patterns of the *o454-nlpD* region

**Fig. 3:**
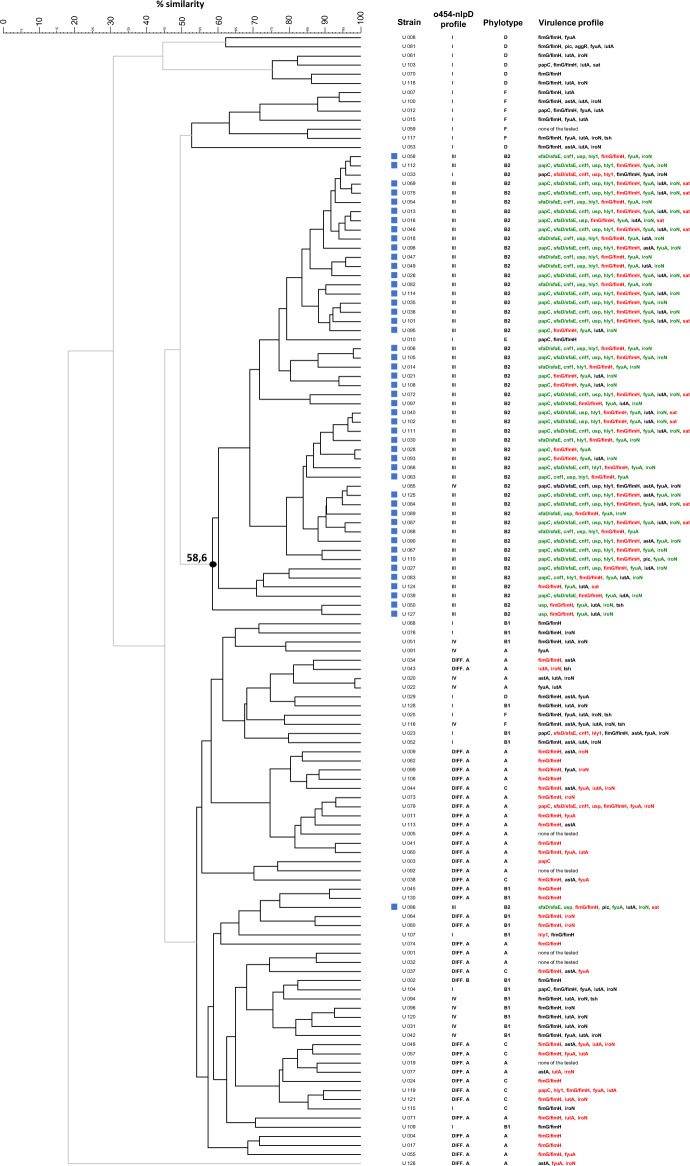
A collective comparative analysis of 124 *E. coli* strains showing clustering in the average CGG-, GTG-, and CAC-PCR fingerprint analyses and the relationships between the *o454-nlpD* profile, phylotype, and virulence factor repertoire

A collective comparative analysis showing the clustering of *E. coli* strains in the averaged TRS-PCR analysis and the relationships between the *o454-nlpD* profile, phylotype, and virulence factor repertoire are presented in Fig. 3.

The cluster grouping mainly highly pathogenic phylogroup B2 and type III *o454-nlpD* strains is marked with a black dot. Strains with profile III *o454-nlpD* are marked with a blue square; pathogenicity factors with a positive correlation with the *o454-nlpD* profile type are marked in green; pathogenicity factors with a negative correlation to the *o454-nlpD* profile type are marked in red.

## Discussion

Extraintestinal infections due to *E. coli* strains constitute an urgent public health problem even though the knowledge about such strains is widened every subsequent year. The genomic plasticity of *E. coli* caused by mobile genetic elements such as PAIs, transposons, phages, and plasmids, which are responsible for encoding various virulence factors, is crucial in adequately classifying this species [28]. Frequent changes in *E. coli* genomes, such as rearrangements, deletions, and insertions, are constantly described [28]. Due to this extreme genome plasticity, new hybrid strains of *E. coli* may occur [24, 28, 29]. Keeping that in mind and the presence of a wide variety of virulence factors, it is not easy to anticipate how pathogenicity might evolve in the *E. coli* population [29]. On the other hand, one of our studies showed that it was possible to predict how pathogenicity might develop in the *E. coli* population [30]. Therefore, studies examining genes related to virulence potential, monitoring the pathogenic capabilities of isolates, and improving valuable diagnostics and epidemiological methods are still of great importance [24].

Our previous research has repeatedly proven the effectiveness of the TRS-PCR test in epidemiological investigations. We demonstrated the ability of this method to detect highly pathogenic *E. coli* strains with multiple VFs [12, 25, 26]. In this study, we decided to compare our previous results for a collection of UPEC strains with those obtained for the same collection regarding the *o454-nlpD* genomic region polymorphism. As changes in the composition of the *o454-nlpD* region and the genetic variability of the *mutS-rpoS* chromosomal region in ExPEC strains are correlated with their virulence [2], we wanted to analyze what type of association for these results we would observe.

Considering statistical analyses of types of *o454-nlpD* region and tested virulence factors, we may state that UPEC strains with pattern IV had no association with determined virulence factors. For UPEC strains with a pattern different A or pattern I, the association with virulence factors was mainly negative, or there was none. Our study demonstrated, however, a positive association between virulence factors such as *papC*, *sfaD/sfaF*, *cnf1*, *usp*, *hly1*, *fyuA*, and *iroN* only for UPEC strains with pattern III. All these pattern III strains were of the B2 phylotype, following the results of Ewers et al. [2]. Moreover, we agree that screening the *o454-nlpD* structure may enable the selection of the most virulent strains from a tested collection of samples, especially for ExPEC and, precisely, UPEC strains [2, 24]. We also showed that the averaged TRS-PCR test (CGG-PCR, GTG-PCR, and CAC-PCR) and the *o454-nlpD* region polymorphism test successfully detected the most virulent *E. coli* strains (Fig. 3).

Noticeably, our analysis of the composition of the *o454-nlpD* region found 38 strains with a pattern not yet described. An amplicon of 1660 base pairs was detected in 37 strains, and one strain had an amplicon of approximately six kbp. The bioinformatic analysis conducted using the appropriate primers (F5 and R2, [2]) and online software (http://insilico.ehu.es/PCR/) enabled us to find complementary regions in the tested *E. coli* genomes. Thanks to this and performed restriction analyses, we confirmed the atypical *o454-nlpD* pattern for this region with a length of approximately 1660 bp, and we named it different A. This pattern was present in 30% of our UPEC strain collection. These thirty-seven strains belonged mainly to phylogenetic groups A (59%) but also C (22%) and B1 (11%). Their virulence profiles were relatively poor, with practically no typical features for uropathogenic strains. In their study, Ewers et al. [2] stated that they observed amplicon sizes other than those described rarely and treated them as an exception.

Moreover, our study identified one strain (U 002) with a different product size than the described amplicons of the *o454-nlpD* region. Its length was approximately 6000 bp, and we named it a different B. This finding should be tested further to recognize the structure of its *o454-nlpD* region correctly and to assess whether it is an exception or potentially new pattern. Strain U 002 belonged to phylogenetic group B1 and, except FimG/H, had none of the tested virulence factors. To avoid discrepancies or misinterpretation, we did not involve this strain in the statistical calculations (Tables 1 and 2).

## Conclusions

To summarize our comparative analyses, the averaged analysis of TRS-PCR band profiles grouped most uropathogenic *E. coli* strains possessing the type III *o454-nlpD* region and, consequently, strains with high pathogenicity potential.

### Supplementary Information

**Additional file 1: Figure S1.** HaeIII restriction analysis of the 1600 bp PCR product of amplification of *o454-nlpD*region for chosen UPEC strains (M1—GeneRulerTM1kb Plus DNA Ladder [Fermentas, Thermo Scientific Waltham, MA, USA); M2—GeneRulerTM50bp DNA Ladder (Fermentas, Thermo Scientific Waltham, MA, USA)].

## Data Availability

Not applicable.
