# Supplementary material for: TRS-PCR profiles correlate with polymorphisms of the genomic o454-nlpD region, virulence factors repertoire, and phylogenetic groups among uropathogenic Escherichia coli strains isolated from patients from Lodz region, Poland
Source: Gut Pathog. 2024 Feb 23;16:11. doi: 10.1186/s13099-024-00603-1 (PMC10885528; doi:10.1186/s13099-024-00603-1)
Supplement: Supplementary file 1 — Additional file 1: Figure S1. HaeIII restriction analysis of the 1600 bp PCR product of amplification of o454-nlpDregion for chosen UPEC strains (M1—GeneRulerTM1kb Plus DNA Ladder [Fermentas, Thermo Scientific Waltham, MA, USA); M2—GeneRulerTM50bp DNA Ladder (Fermentas, Thermo Scientific Waltham, MA, USA)]. [file 13099_2024_603_MOESM1_ESM.pdf]

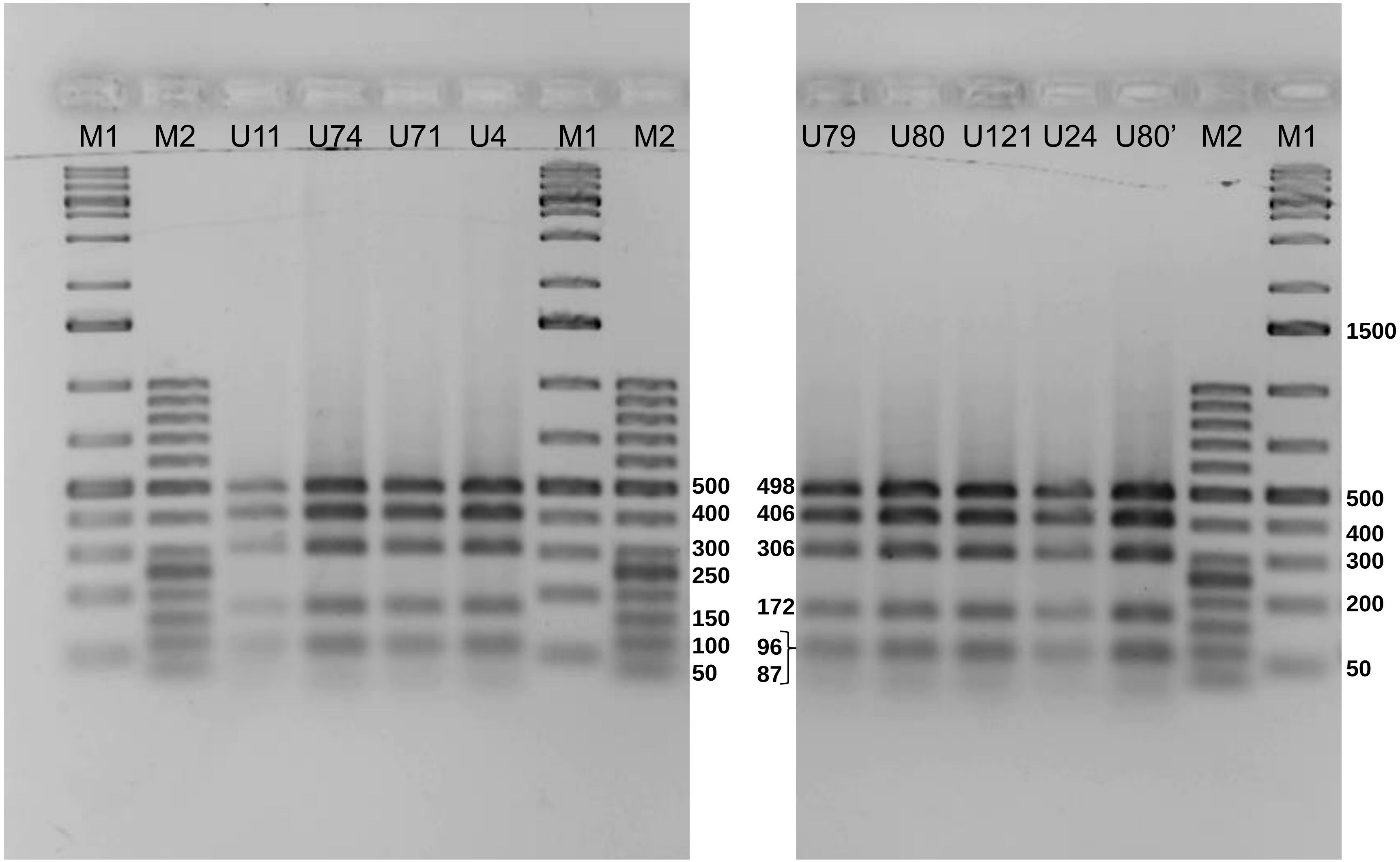

Fig. 1S. Hae III restriction analysis of the 1600 bp PCR product of amplification of *o454-nlpD* region for chosen UPEC strains (M1 – GeneRuler™ 1kb Plus DNA Ladder (Fermentas, Thermo Scientific Waltham, MA, USA); M2 – GeneRuler™ 50bp DNA Ladder (Fermentas, Thermo Scientific Waltham, MA, USA)).
